# Novel pH-Responsive Cubosome and Hexosome Lipid Nanocarriers of SN-38 Are Prospective for Cancer Therapy

**DOI:** 10.3390/pharmaceutics14102175

**Published:** 2022-10-12

**Authors:** Sarigama Rajesh, Jiali Zhai, Calum J. Drummond, Nhiem Tran

**Affiliations:** School of Science, STEM College, RMIT University, Melbourne, VIC 3000, Australia

**Keywords:** pH-responsive, cubosomes, hexosomes, lipid-nanoparticles (LNPs), SN-38, ionisable lipids, aminolipid, MO, drug delivery, self-assembly, lyotropic liquid crystals

## Abstract

**Highlights:**

1) **What are the main findings?**
  - Lipid nanoparticles containing novel synthetic aminolipids were formulated. Their internal nanostructures are sensitive to pH, being an inverse hexagonal phase at pH 7.4 and a bicontinuous cubic phase at pH 4.0–pH 7.0.
  - Poorly soluble and highly potent anticancer drug SN-38 was encapsulated successfully in the nanoparticles with a ~100-fold increase in solubility.
  - SN-38 was released faster at a tumour-relevant acidic pH compared to neutral pH.
2) **What is the implication of the main finding?**
  - Aminolipids can be incorporated into self assembled lipid nanoparticles to achieve desirable pH responsiveness.
  - The SN-38-loaded pH-sensitive lipid nanoparticles represent a promising candidate for delivering the potent drug SN-38 to tumour sites.
  - The pH-sensitive lipid nanoparticles in this study can be a viable alternative to the currently used irinotecan.

**Abstract:**

pH-responsive nanoparticles enable the selective delivery of a chemotherapeutic agent to tumours while reducing adverse effects. Herein we synthesised four novel aminolipids and developed pH-responsive nanostructured lipid nanoparticles (LNP), which exhibited a slow-releasing hexagonal structure (H_2_) at physiological pH and quick release bicontinuous cubic phase (Q_2_) at the acidic tumour pH. The nanoparticles were used to encapsulate and control the release of the chemotherapeutic agent SN-38. High-throughput formulation techniques were employed to fabricate LNP by mixing various amounts of aminolipid with monoolein (MO). The effect of aminolipids on MO self-assembled structures was studied using small-angle X-ray scattering (SAXS) at various pH values. Out of the four studied aminolipid-MO LNP systems, the nanoparticles containing N-(Pyridin-4-ylmethyl) oleamide (OAPy-4) or N-(2(piperidin-1yl)ethyl) oleamide (OAPi-1) exhibited a pH-induced H_2_ to Q_2_ phase transition in a tumour-relevant pH range (pH 5.5–7.0). SN-38 is 1000 times more efficacious than the commercially available prodrug irinotecan. However, low solubility in water and instability at physiological pH makes it unsuitable for clinical use. SN-38 was loaded into LNP containing MO and aminolipid OAPy-4. The drug loading and entrapment efficiency were determined, and the results indicated that the aqueous solubility of SN-38 loaded in LNP dispersions was ~100 times higher compared to the solubility of the pure drug in aqueous solution. Furthermore, we demonstrated that the in vitro SN-38 release rate from LNPs was faster at lower pH (pH 5) than at neutral pH. Therefore, pH-responsive LNPs developed in this study can potentially be employed in delivering and controlling the release of the potent drug SN-38 to tumour sites.

## 1. Introduction

**Figure 1:**
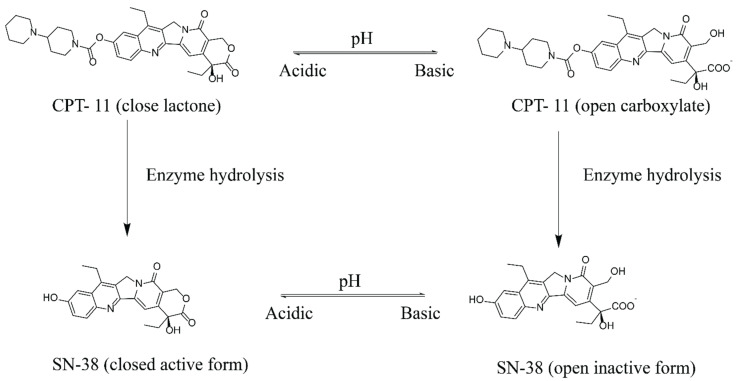
The active form (lactone) chemical structure of Camptothecin derivate CPT-11 and its enzyme hydrolysis to SN-38 and its pH-dependent inactive carboxylate form.

SN-38 is an anticancer drug belonging to the naturally occurring alkaloid family called camptothecin (CPT). SN-38 blocks the enzyme responsible for DNA replication, thereby inducing cell death. A key advantage of SN-38 is that it is 100–1000 times more potent than the commercially available water-soluble derivative irinotecan (CPT-11). However, SN-38 exhibits extremely low solubility in water (7–11 μg/mL), and most of the other physiologically compatible solvents, such as ethanol (<1 mg/mL), making it practically impossible to formulate and limit its clinical use. The bipiperidine ester prodrug of SN-38, CPT-11 or irinotecan, which is soluble in water, is currently used in clinics to treat colorectal cancer [1]. After administration, the carboxylesterase enzymes in the liver metabolise the CPT-11 to SN-38 (Figure 1). Studies have suggested that the conversion rate of the prodrug CPT-11 to SN-38 is meagre (around 2–8%) [2,3].

Drug instability is another significant barrier to developing an effective SN-38 therapy. At physiological pH (pH 7.4), SN-38 will be in its open carboxylate form, which shows no therapeutic effect. Furthermore, the active form of SN-38 is only stable at pH ≤ 4.5, and, with an increasing pH, the molecule undergoes hydrolysis with a structural change from a closed active lactone ring to an open inactive carboxylate form. As represented in Figure 1, both active and inactive forms are in equilibrium at pH 6.7.

Several groups have employed a nanomedicine encapsulation approach to overcome the limitations of ultralow solubility and poor stability of SN-38 at physiological pH. Lipid nanoparticles, including liposomes, solid–lipid nanoparticles, micelles, and nanoemulsions were developed to protect and deliver SN-38 [4,5,6,7]. Monterrubio et al. created SN-38-loaded nanofibre matrices with 18 μg SN-38 per cm^2^ for localised delivery to solid tumours [8]. The local release of SN-38 from microcrystals is in high concentration and penetrates a distance of 2 mm within the solid tumour [8]. Ranneh et al. loaded the SN-38 in phytantriol-based cubosomes using surfactants to enhance the solubility and stability and achieved a high loading formulation of SN-38 of 90–120 µg/mL using cationic surfactants [9]. Recently, Feifei et al. also demonstrated that the nanoformulation of SN-38 exhibits lower hepatotoxicity than its sole-molecule counterpart [10].

A recent review by Si et al. summarised different strategies and development approaches for the delivery of SN-38 [11]. However, the encapsulation efficiency and total quantity of SN-38 in the nanoparticles are still relatively low, and preparing a consistent formulation is still extremely challenging due to ultralow solubility. This low drug loading has elicited some research interest in converting SN-38 into lipophilic prodrug entities [12]. The prodrug of SN-38 can dissolve in some organic solvents such as dichloromethane or ethanol and then be encapsulated into a nanoparticle form with improved loading [13,14,15]. Wang et al. recently synthesised peptide–drug conjugated prodrug, which self-assembled into nanostructures. The optimised SN38–peptide prodrug formed a stable micelle with a hydrodynamic diameter of ~110 nm and drug loading as high as 35% [16]. However, the prodrug approach can add complexity in meeting regulatory requirements.

Lyotropic liquid crystalline (LLC) lipid nanoparticles (LNP) are colloidal systems consisting of amphiphiles that can give rise to various nanostructures when self-assembled in water [17]. Depending on the effective geometry of the amphiphile molecules, nanostructures such as the lamellar (L_α_) and nonlamellar mesophase, including bicontinuous cubic (Q_2_), hexagonal (H_2_), or micellar cubic (I_2_), can be formed [18]. The bulk lipid–water mesophase can be dispersed in the presence of a steric stabiliser to form nanoparticles. When dispersed in an aqueous solution, these nanoparticles retain their internal nanostructures and are called liposomes, cubosomes, hexosomes, and micellar cubosomes, respectively. Many past studies have demonstrated that the drug release rate from these amphiphile self-assembled structures is governed directly by the structures themselves [19,20]. Internal nanostructures of these LNP also appear to affect cell uptake, haemolysis, and cytotoxicity [21,22,23]. Generally, cubosomes with an internal bicontinuous cubic structure are quicker to release drugs and exhibit stronger cell membrane fusion than hexosomes and other mesophases [24,25,26]. Recently, several studies demonstrated a pH-responsive LNP system with slower-releasing hexosomes at blood pH (7.4) and fast-releasing cubosomes at tumour pH (5.5–6.5) [25,27].

The pH-responsive phase transition in LLC LNPs can be manipulated by doping an ionisable amphiphile to the lipid matrix. The ionisation state (protonation/deprotonation) of the added amphiphile causes an alteration in lipid packing, which drives a phase transition. Negrini et al. were the first to demonstrate the phase transition from H_2_ to Q_2_ as pH decreased from 7.4 to 5.0 by adding a synthetic aminolipid ester, pyridinylmethyl linoleate, to bulk lipid monolinolein [28]. Their results showed a tenfold faster release of doxorubicin at pH 5 while being threefold more effective in its anticancer activity than at pH 7.4. Recently, our group reported the synthesis of a library of nine aminolipids, also with an ester linker, which were successfully formulated with MO into pH-responsive nanoparticles [29]. These particles underwent a phase transition from H_2_ at neutral pH to Q_2_ at lower pH (pH 4–7), making them prospective candidates for delivering chemotherapeutic drugs [29].

**Figure 2:**
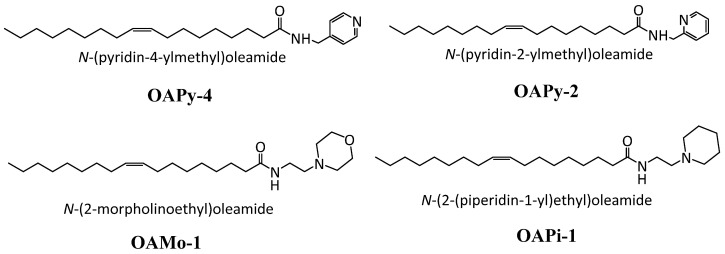
The chemical structure of amide ionisable aminolipids synthesised by employing a coupling reaction between the oleic acid and primary amines.

The current study investigated pH-responsive LLC nanoparticles as nanocarriers for SN-38. First, we synthesised four new ionisable aminolipids, which contain an oleyl tail connected to a headgroup bearing tertiary amines via an amide linker (Figure 2). An amide linker is less prone to hydrolysis compared to an ester linker; thus, the aminolipids in this study will be more stable than previously reported esters. Next, these aminolipids were incorporated into MO-based nanoparticles, and the mesophase behaviour of the formulations at pH 3–8 was determined using synchrotron small-angle X-ray scattering (SAXS). The effect of adding amide aminolipids on MO self-assembled structures was studied by determining partial phase diagrams using the SAXS data for each of the four LNP systems. SN-38 was then loaded into specific pH-responsive LNPs, and the entrapment efficiency, drug loading, and in vitro drug release were measured. This study represents remarkable progress in developing pH-responsive chemotherapeutic drug delivery vehicles for highly hydrophobic drugs such as SN-38.

## 2. Materials and Methods

### 2.1. Materials

4-(Aminomethyl) pyridine, 2-picolylamine, 4-(2-aminoethyl) morpholine, 1-(2-aminoethyl) piperidine, N-(3-dimethylaminopropyl)-N′-ethyl carbodiimide hydrochloride (EDC), hydroxybenzotriazole (HOBt), dimethyl aminopyridine (DMAP), dichloromethane (DCM), n-hexane, ethyl acetate, sodium sulphate, sodium chloride, deuterated chloroform, deuterated dimethyl sulfoxide, deuterium oxide, and Pluronic F-127 were purchased from Sigma-Aldrich. For all aqueous preparations, Milli-Q water was used. Monoolein (MO) and oleic acid (OA) were procured from Nu-chek Prep (GC > 99%), and SN-38 was obtained from Adooq bioscience.

### 2.2. Synthesis of Ionisable Aminolipids

Ionisable amide-aminolipids were synthesised via a coupling reaction between oleic acid (OA) and amines. The procedure followed for the coupling reaction was similar to our previously published procedure [29]. In brief, an equimolar solution of amine in DCM was slowly added to cooled solution of OA containing EDCl (1.1 eq.) and DMAP (0.25 eq.). For reaction completion, the resulting reaction mixture was stirred for 24 h at RT (room temperature). The resulting crude mixture after removing the solvents was purified using a silica column. The purified aminolipid was analysed using nuclear magnetic resonance (NMR) spectroscopy, which confirmed the chemical structure.

### 2.3. Lipid Nanoparticle Fabrication

LNPs were prepared using a high-throughput formulation by mixing aminolipids with MO at various ratios. Aminolipids were mixed at an incremental ratio to the organic phase containing MO. R_AL_ indicates the ratio of the added aminolipids to the total amount of lipids in the system [29]. Aminolipids were mixed at ratios of 95:5, 90:10, 85:15, 80:20, 75:25, 70:30, 60:40, and 50:50 to MO, corresponding to R_AL_ values of 0.05, 0.1, 0.15, 0.2, 0.25, 0.3, 0.4, and 0.5, respectively. The organic solvent was removed completely, and then 1 mL of F-127 (2 mg/mL) dissolved in DI water was added to this dried lipid mixture. The resulting mixture was probe-sonicated (Q Sonica) at the output power of 35 W, with a pulse of 5 s on/5 s off for a total of 5 min to obtain milky opaque solutions.

The drug encapsulated formulations were obtained by first preparing the dry lipid mixture and then adding solid SN-38 to the lipid mixture, before heating the resulting mixture at 60 °C overnight. The resulting lipid melt was mixed using a vortex mixer, and then an aqueous solution of F-127 was added. A probe sonicator was used to disperse the bulk lipid to obtain the LNPs [9].

### 2.4. Particle Size Distribution of LNP

The LNP dispersions were characterised for their average hydrodynamic diameter (in nm), and polydispersity index (PDI) was measured using a Malvern Zetasizer Nano ZS (Malvern Instruments, Malvern, Worcestershire, UK). All measurements were performed at RT (25 °C and n = 3), and measurements were performed after diluting LNPs with Milli-Q water at a ratio of 95:5 [30].

### 2.5. Synchrotron SAXS Characterisation of LNP

The high-throughput SAXS analysis was similar to the procedure reported in our previous study, wherein the LNPs were diluted with a suitable buffer solution of specified pH [29]. Briefly, 40 μL of LNP and 40 μL of pH buffer solutions, prepared using citric acid and sodium phosphate (pH 3–pH 10), were added to a transparent 96-well plate. Liquid crystalline structures of each of the LNPs were characterised in 12 different pH conditions. Experiments were conducted at the SAXS/WAXS beamline of the Australian Synchrotron, and the scattering patterns were obtained at RT and 37 °C [20,29,31].

### 2.6. Drug Loading and Encapsulation Efficiency (EE%)

The nanoparticle dispersions were centrifuged at 1000× *g* for 20–25 min at RT to remove any unencapsulated drug. The supernatant was collected and vortexed for 2 min at 2500 rpm for uniform distribution. Drug loading and %EE were determined using the HPLC method reported in our previously published journal article [32]. In brief, HPLC analysis was conducted by employing the C18 column (4.6 mm × 250 mm, 5 μm), and the column temperature was maintained at 30 °C during the run. An isocratic mixture (50:50 *v*/*v*) of 25 mM sodium phosphate buffer solution (adjusted to pH 3.1) and acetonitrile was used as the HPLC solvent; during an assay, the flow rate was maintained at 1 mL/min. The HPLC samples were prepared by diluting the LNPs with the HPLC solvent, in a ratio of 1:99 with an injection volume of 20 μL. A standard curve was drawn using 0 μg/mL, 1 μg/mL, 5 μg/mL, 10 μg/mL, 20 μg/mL, and 40 μg/mL samples, and the SN-38 content in the LNPs was quantitatively determined using the standard curve. LNPs were analysed for SN-38 content before (Wi) and after (Wa) the centrifugation step.

The following equation was used to calculate the encapsulation efficiency (EE%):

(1) $$EE\;\%=\frac{\mathrm{Wa}}{Wi}\times 100.$$

### 2.7. Cryogenic Transmission Electron Microscopy (Cryo-TEM)

The visualisation of the selected representative nanoparticles was carried out using cryo-TEM. For this, copper grids (200-mesh) coated with perforated carbon film using a FEI Vitrobot were glow discharged in nitrogen to render them hydrophilic and placed in a laboratory-built humidity-controlled vitrification system. Aliquots of samples were placed on the grids and after 30 s adsorption time, grids were blotted manually using filter paper for approximately 3 s.

Grids were then plunged into liquid ethane cooled by liquid nitrogen. The samples were examined using a FEI Tecnai 12 Transmission Electron Microscope operating at 120 kV at −190 °C, at a defocus level of 1.5–2 μm, with an electron dose of <900–1000 electrons nm^−2^.

### 2.8. Drug Release Study

The in vitro release of SN-38 from the nanoparticles was evaluated using a dynamic dialysis method [33]. After removing unencapsulated drug, 1 mL of SN-38-loaded nanoparticles was placed inside a dialysis tube (Pur-A-Lyzer™ Maxi Dialysis tube molecular weight cut-off 10 kDa) and immersed in 1000 mL of phosphate buffer maintained at 37 ± 1.0 °C and stirred at 400 rpm. Release experiments were performed in triplicate and on three independently prepared SN-38 encapsulated LNPs. For physiological pH, experiments were carried out by dialysing the formulations against 1000 mL of phosphate buffer (pH 7.0). Lower pH experiments were conducted by dialysing the formulations against 1000 mL of phosphate buffer adjusted to the required pH. The HPLC method quantitatively measured the SN-38 that remained in the dialysis tube. At predetermined time intervals (60 min, 120 min, 240 min, and 420 min), 10 µL of the sample was withdrawn from the dialysis tube and diluted with 990 µL of the HPLC solvent [7]. The method of determining the release kinetics by drawing the sample from inside the dialysis bag was discussed in detail in recent papers [34,35,36,37].

## 3. Results and Discussion

### 3.1. Synthesis of Ionisable Aminolipids

Synthesis of aminolipids was conducted by coupling oleic acid with primary amine containing an ionisable tertiary amine group. The crude compound after the reaction was purified using a silica column to remove the excess reagent and unreacted starting material. The reaction yield was in the range of 50–70%. NMR results indicated that the lipids were relatively pure without any identifiable impurity. The physical state of the synthesised amide-aminolipids was semisolid at RT, with a reaction yield of 70–85%. The structural confirmation was determined using NMR, and the data are provided in Supplementary Figure S1.

### 3.2. Lipid Nanoparticle Fabrication

Aminolipids were mixed with MO at an incremental ratio from 5 wt.% to 50 wt.%. To stabilise the nanoparticles, Pluronic F-127 was used, and the amount of F-127 was kept at 10 wt.% with respect to the total lipids in the system. These formulations were all well dispersed with no visual sedimentation or phase separation. Specifically, each formulation was characterised by an average particle size and PDI. These values are presented in Table 1 and Table 2. The particle size range was 137–300 nm, and the PDI range was 0.15–0.3.

These particle size and PDI values are in an expected range for MO-based LNPs, and similar particle size ranges have been observed in previous studies [29,30]. To evaluate the stability of LNPs at RT, particle sizes were also measured after 30 days of standing at RT. There were no significant changes in their size and the physical appearance of the LNPs, implying that the prepared LNPs were stable for the tested time of 30 days (data not included).

### 3.3. Synchrotron SAXS Analysis

Aminolipid-containing MO LNPs were studied using synchrotron SAXS to determine their phase behaviour. The aminolipids were added at eight different compositions, corresponding to ratios of aminolipid to total lipid R_AL_ of 0.05, 0.1, 0.15, 0.2, 0.25, 0.3, 0.4, and 0.5. The mesophase structure of each of these LNP systems was determined for at least 10 individual pH values (in the pH range of 3.0 to 10).

**Figure 3:**
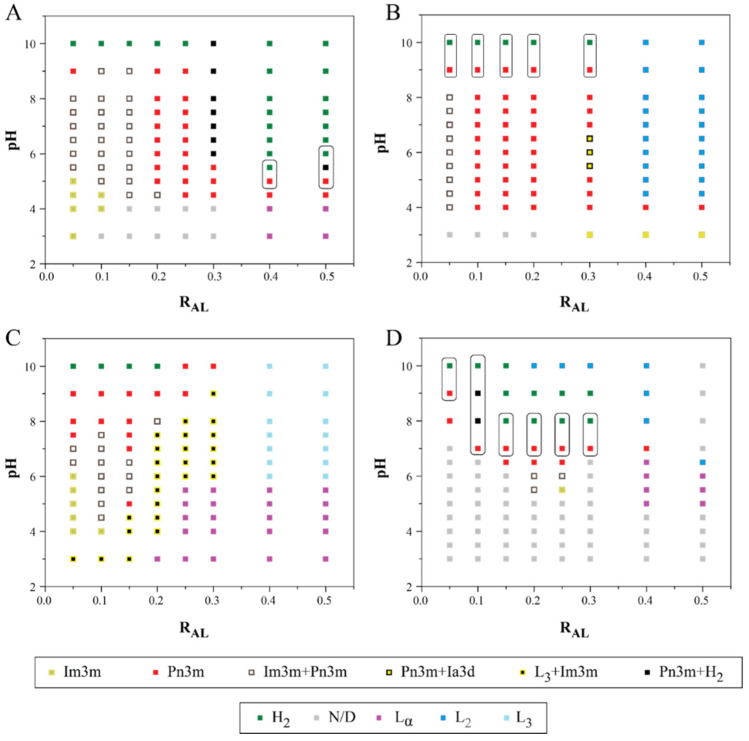
SAXS derived partial phase diagrams at RT for LNPs prepared by adding aminolipid to MO at pH 3–10. The boxes highlight the desired hexagonal (H_2_) to cubic (Q_2_) phase transition as pH drops. Partial phase diagrams for nanoparticles obtained by incorporating (**A**) OAPy-4, (**B**) OAPy-2, (**C**) OAMo-1, and (**D**) OAPi-1. R_AL_ represents the weight ratio of aminolipid to the total weight of lipids in the system. Identifiable phases include bicontinuous cubic (Im3m, Pn3m, and Ia3d), sponge phase (L_3_), inverse micelles (L_2_), hexagonal (H_2_), and lamellar (L_α_). N/D indicates a nonidentifiable phase.

The formation of various mesophase structures can be rationalised through the concept of the critical packing parameter (CPP); CPP = *V*/*al*, where *V* is the effective volume of the amphiphile’s hydrophobic tail, *a* is the headgroup’s effective area, and *l* is the effective length of the hydrophobic chain. Lamellar phases (L_α_) are typically observed with a zero interfacial curvature (CPP = 1). For CPP values greater than 1, inverse phases, such as Q_2_ and H_2_, are formed with increasing interfacial curvature. Q_2_ phases consist of a single continuous curved lipid bilayer creating an ordered three-dimensional complex cubic network, separating two nonintersecting but continuous water channels [38]. The three main types of cubic structures observed in amphiphile–water systems are gyroid (G), diamond (D), and primitive (P) surfaces, which correspond to space groups Ia3d (G), Pn3m (D), and Im3m (P), respectively. An H_2_ phase displays an ordered two-dimensional structure with water channels surrounded by a lipidic bilayer (reversed cylindrical micelles), which are packed on a hexagonal array [39,40]. An increase in factors such as temperature, hydrophobic chain unsaturation, tail length, and tail branching generally results in higher CPP. Figure 3 displays partial phase diagrams with pH and R_AL_ varied for all LNPs at RT. The change in the mesophase structure of LNPs due to the addition of aminolipids at neutral pH (concentration effect) and changing pH (pH effect) is discussed in detail below.

#### 3.3.1. Effect of Lipid Composition on Mesophase Structure of Aminolipid Doped MO-LNPs

MO forms a cubic phase with excess water in a wide range of concentrations and temperature and is largely used to prepare cubosomes at ambient temperatures [41,42]. MO stabilised with F-127 at RT in excess water exhibited an Im3m cubic phase [24,43]. At neutral pH, adding an increasing amount of OAPy-4 and OAPy-2 to MO LNP induced a phase transition of Q_2_ → H_2_ → L_2_ (Figure 3A,B). For MO doped with OAPi-1, at neutral pH for R_AL_ = 0.1–0.4, a Pn3m cubic structure was observed (Figure 3D). Considering that MO LNPs without any aminolipid exhibit an Im3m cubic phase, the transition into a Pn3m structure suggested that the addition of OAPi-1 also increased the lipid surface curvature, similar to the case of OAPy-4- and OAPy-2-doped MO (at pH 7). The sequential change can be rationalised through the changes in the effective molecular packing parameter (CPP). MO has two –OH (hydroxyl) groups as part of its headgroup, and hydroxyl groups can form hydrogen bonding, which results in MO having an effective larger headgroup size than OAPy-4 and OAPy-2. Adding OAPy-4 and OAPy-2 to MO, therefore, further increases the effective CPP and leads to the formation of the more curved hexagonal phase. This trend is similar to that observed in our ester aminolipid-doped study and other previous studies, where more hydrophobic molecules such as fatty acids and oils were added to the MO cubic phase [29,31,44].

The doping concentration at which the phase transition from Q_2_ to H_2_ occurs depends on the lipophilicity of the doped molecule and its ability to form hydrogen bonding either with the adjacent lipid or with water. For example, for nanoparticles containing OAPy-4, the transition from Q_2_ to H_2_ occurs at R_AL_ = 0.4. The doped aminolipid OAPy-4 is very similar to Lipid-1 studied in our previous work, with the only molecular difference being the linker (i.e., amide vs. ester) [29]. Log P values for Lipid-1 and OAPy-4 are 6.95 and 6.12, respectively (theoretical values obtained from Chemdraw), indicating that Lipid-1 is more lipophilic [45]. Interestingly for OAPy-4-doped MO LNPs, the Q_2_ to H_2_ transition at neutral pH occurred at a much higher lipid concentration than for the Lipid-1-containing system (R_AL_ = 0.15). This may be due to the higher hydrophilicity and ability of OAPy-4 to form hydrogen bonding compared to its ester analogue Lipid-1.

Surprisingly, for OAMo-1, an opposite trend was observed upon adding an increased amount of OAMo-1 to MO LNPs at neutral pH. The phase change sequence was bicontinuous cubic → (mixed cubic + sponge) → sponge (Q_2_ → Q_2_ + L_3_ → L_3_). The L_3_ phase was found in a very narrow range between L_α_ and Q_2_ and can be described as a melted cubic phase without having a long-range order. The co-existence of the sponge phase and Q_2_ phase was initially observed at R_AL_ = 0.2–0.3 (pH 7) (Figure 3C). Further addition of OAMo-1 resulted in a single sponge phase (R_AL_ > 0.4, pH 7). This difference is attributed to the chemical structure of OAMo-1, with an amide linker attached to hydrophilic morpholine, making the effective hydrophilic headgroup area larger than that of MO. Adding a molecule which is more polar or has a larger head group size than MO could induce less curvature in the lipid membrane, thereby decreasing the effective CPP. A similar trend was observed by Gontsarik et al., while adding antimicrobial peptide to MO cubosomes, where the internal phase transformed in the order Q_2_ → L_3_ → L_α_ → micelles with increasing peptide concentration [46]. LNPs were also prepared using 100% aminolipid (without any MO) and stabilised with F-127, whereby the SAXS data indicated the formation of inverse micelles (L_2_ phase) with no ordered mesophase structure found (data not included).

#### 3.3.2. Effect of pH on Mesophase Structure of Aminolipid-Doped MO LNPs

**Figure 4:**
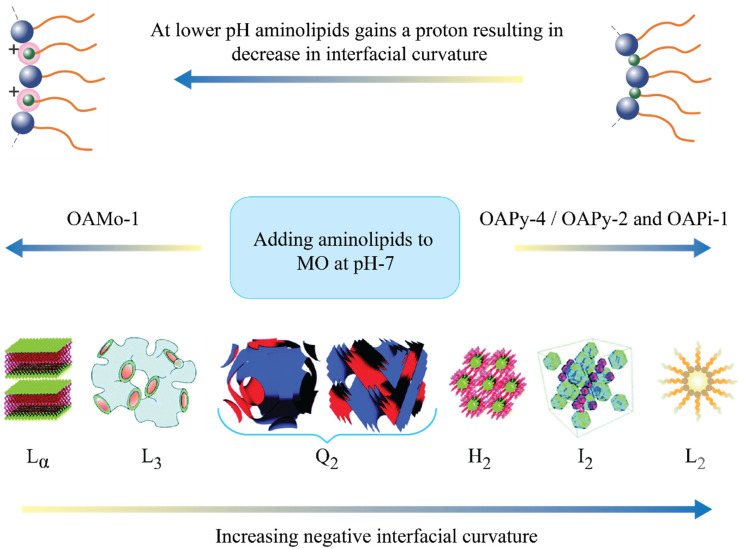
Graphical representation of the concentration effect observed for LNPs prepared by adding an increasing amount of aminolipids to MO (at pH 7). For OAPy-4, OAPy-2, and OAPi-1, the observed phase transition was Q_2_ → H_2_ → L_2_ while the phase transition was Q_2_ → Q_2_ + L_3_ → L_3_ for nanoparticles containing OAMo-1. Changing pH can result in a change in ionisation of the aminolipids and interfacial curvature; with decreasing pH, the transition followed the sequence L_2_ → H_2_ → Q_2_ for all four studied aminolipids.

The influence of pH on the mesophase structure of LNPs containing MO and aminolipids was studied in a pH range from 3.0 to 10.0. Their pH- and composition-dependent partial phase diagrams are shown in Figure 3. LNPs containing only MO and stabilised with F-127 displayed an Im3m cubic phase, which was independent of the pH change between pH 3 and pH 8. From Figure 3, a general observed trend for aminolipid-doped MO LNPs is that, as the pH of the system became acidic, the interfacial curvature decreased, leading to phase transformation to structures with lower CPP (e.g., H_2_ to Q_2_). This observation is similar to our previous study [29], wherein it was confirmed that the ionisation of the aminolipids is the fundamental reason for the pH responsiveness of the LNPs. The aminolipid headgroup contains tertiary amines, which gain a proton at an acidic pH along with acquiring a positive charge. The combination of a headgroup acquiring a proton and charge–charge electrostatic repulsion results in the expansion of the headgroup size and a decrease in CPP. As depicted in Figure 4, at lower pH, the sequence in which the phase transition occurred was L_2_ → H_2_ → Q_2_ (CPP high to low).

The pH range in which a phase transition occurs depends on many factors such as the molecular structure of the aminolipid, amount doped, and its apparent pK_a_ (pK_a_^app^) [47]. In an aminolipid-doped MO LNP system, the ionisable aminolipid molecules reside at the lipid–water interface, and the aminolipid acquiring a positive charge with pH depends on its pK_a_^app^ at the lipid–water interface. This pK_a_^app^ differs from that of aminolipid alone dispersed/dissolved in water (pKa^w^) and depends on the surface charge density at the interface, ionic strength of the medium, and medium effect, which is a result of the interfacial microenvironment [47]. It should also be noted that, due to the complexity of the dispersed LNPs systems, the R_AL_ and pH values at which the phase transition occurred could not be predicted a priori.

For cancer drug delivery, as mentioned previously, a phase transition from a slow release H_2_ phase to a fast release Q_2_ phase is desirable. For OAPy-4-containing LNPs, the desired phase transformation from H_2_ to Q_2_ was observed at pH 7.5 **→** 5.5 in a concentration range R_AL_ = 0.4–0.5. For LNPs with OAPi-1, at R_AL_ = 0.2–0.3, the desired H_2_ → Q_2_ transition was observed at pH 7.0, within a narrow range of 0.5 pH unit difference. For the same system at an R_AL_ of 0.05, the H_2_ → Q_2_ transition occurred with the decrease in pH but at a much higher pH value (pH 8–10). For systems containing OAPy-2 and OAMo-1, no H_2_ → Q_2_ transition was detected. For LNPs with OAPy-2 at R_AL_ = 0.4–0.5, an L_2_ to Q_2_ transition was observed at pH 3.0. For LNPs with OAMo-1, lowering the pH resulted in mixed L_3_ and weakly ordered Q_2_, while further reduction in pH resulted in an L_α_ phase. It should be noted that, for LNPs with OAPi-1, there were a large number of pH points, hindering the identification of mesophases, due to the poor scattering nature of the peaks, and they are denoted as N/D in the partial phase diagram.

Cryo-TEM images of MO cubosomes, MO loaded with OAPy-4 hexosomes at pH 7, and MO loaded with OAPy-4 cubosomes at pH 5 are provided in Supplementary Figure S2. The sizes of the nanoparticles were within the range of the DLS measurements. Their structures also matched SAXS peak assignments.

#### 3.3.3. Effect of Temperature on Mesophase Structure of Aminolipid-Doped MO-LNPs

**Figure 5:**
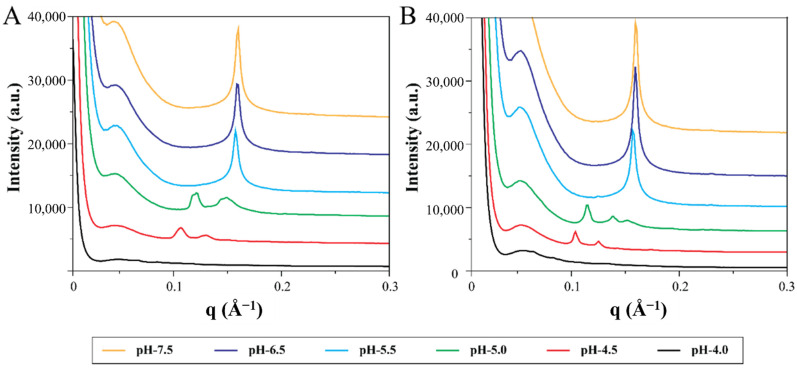
Representative one-dimensional SAXS profiles for nanoparticles prepared by doping OAPy-4 to MO at R_AL_ = 0.4 at 37 °C (**A**) and at room temperature (**B**).

LLC phases are also temperature-dependent; therefore, to evaluate the fate of the nanoparticle at body temperature, an SAXS study of phase behaviour was performed at 37 °C. For this purpose, we selected the formulation with OAPy-4 at R_AL_ 0.4, which exhibited the H_2_ phase at pH 7.5 and a transition to Q_2_ at lower pH. These LNPs exhibited the desirable phase transition (H_2_ → Q_2_) in a disease-relevant pH range for cancer drug delivery. One-dimensional SAXS profiles for nanoparticles at pH 4.0 to pH 7.5 are presented in Figure 5. From the SAXS profile, it is evident that the mesophase structures of these aminolipid-doped LNPs did not change significantly between 25 °C and 37 °C.

### 3.4. Drug Loading

**Figure 6:**
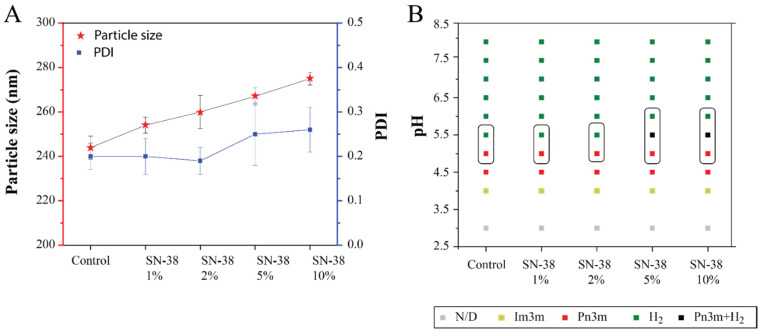
(**A**) Particle size and size distribution (PDI) of SN-38-loaded MO + OAPy-4 LNP. The control sample was LNPs without SN-38. The LNPs were prepared by adding 1, 2, 5, and 10 wt.% of SN-38 to the total quantity of lipid mixture consisting of MO and OAPy-4, which were stabilised by Pluronic F-127. The experiments were performed in triplicate, and the results were reported as the mean ± standard deviation. (**B**) A partial phase diagram for SN-38-loaded LNPs with respect to pH at 37 °C. The boxes represent the phase transition from H_2_ to Q_2_ with the decrease in pH.

The very low solubility of SN-38 in water and most organic solvents constrains the usual procedure to make LNPs using the dry film hydration method. Hence, a modified method was used, wherein a solid powder of SN-38 was added to the melted lipid mixture, which was then heated at 60 °C overnight. An aqueous solution of F-127 (2 mg/mL) was added to the lipid melt, and ultrasonication was used to prepare the LNPs. Four formulations with different quantities of SN-38 were prepared with OAPy-4 and MO (R_AL_ = 0.4). The amount of SN-38 added was 1, 2, 5, and 10 wt.% with respect to the total quantity of lipid mixture in the formulation. Visual observation of the formed LNPs indicated that the formulations were well dispersed, but some precipitate was observed especially at high loading (i.e., 5 wt.% and 10 wt.% SN-38). The physicochemical properties of the SN-38-loaded LNP, including particle size and PDI, are presented in Figure 6A. SAXS experiments were performed to study the internal mesophase structure of SN-38-loaded LNPs in response to pH change. The studies were conducted by varying the pH in the range of pH 3 to pH 8 at 37 °C (Figure 6B).

#### 3.4.1. Physiochemical Properties and Partial Phase Diagram for SN-38-Loaded LNPs

The particle size of the drug-loaded formulation was in a range of 244 nm to 274 nm with a PDI of 0.19–0.26. In general, an increased amount of SN-38 added to the system resulted in a slight gradual increase in the hydrodynamic diameter of the LNPs. LNPs without SN-38 were measured to have a diameter of 244 nm, while LNPs doped with 10 wt.% SN-38 were larger at 274 nm. In previous work, Carsado et al. reported a significant increase in liposomal size for their novel microfluidic SN-38-loaded formulation, and this increase depended on the lipid–drug molar ratio [48]. Carsado et al. developed liposomes containing soybean lipid extract as the lipid component and SN-38; at a lipid–drug molar ratio of 40:1, the particle size was 119.1 ± 1.9 nm, whereas, when the molar ratio was increased to 7.5:1, the liposomal particle size was 355.3 ± 13.2 nm. Similarly, Pedram et al. also saw an increase in the particle size of the SN-38-loaded poly-lactide-*co*-glycolide nanoparticles [49].

Synchrotron SAXS experiments were conducted with the SN-38-loaded LNPs to evaluate the effect of increasing SN-38 content on the mesophase behaviour. All SN-38-loaded formulations behaved slightly differently compared to the LNPs without the drug. For formulations with 1 and 2 wt.% SN-38, the phase transformation H_2_ → Q_2_ occurred at pH 5.0, which was similar to the control without SN-38. However, for LNPs with 5 and 10 wt.% SN-38, co-existence of two phases (Pn3m + H_2_) was observed at pH 5.5, and a complete transformation to a Q_2_ phase occurred at pH 5. The slightly higher H_2_ → Q_2_ transition pH can be explained due to the hydrophobicity of SN-38, as hydrophobic molecules are known to reside in the hydrophobic core of the lipid bilayer [48,50]. Results presented here indicate that the highly hydrophobic drug SN-38 can be encapsulated into LNPs without markedly changing its pH responsiveness or its stability. The influence of molecular species addition to MO cubic phases was recently reviewed [51].

#### 3.4.2. Determining Encapsulation Efficiency

Drug loading (DL%) is the ratio of the quantity of drug in the nanoparticle to the total weight of the nanoparticles. Encapsulation efficiency is the ratio of the encapsulated amount of the drug to the initial amount of the drug added while preparing nanoparticles. The EE% for LNPs loaded with 10 wt.% SN-38 was measured to be 42% ± 14%, which corresponds to an average concentration of 844 µg SN-38 per mL of the formulation (Table 3). The drug loading of 844 µg/mL (DL% of 3.8%) is equivalent to ~100 times the solubility of SN-38 alone in water. A recent study conducted by Ranneh et al. used cationic surfactants to load SN-38 into phytantriol-based cubosomes and achieved a loading of 90–120 µg/mL [9]. Compared to the study conducted by Ranneh et al., our formulations with ionisable aminolipids possessed a drug concentration ~8 times higher. Many factors contribute to the drug loading, including the lipid composition, the process of adding the drug, and the bilayer fluidity [52,53]. A similar high loading of the drug SN-38 was observed in our recent study with MO cubosomes stabilised by amphiphilic lipid poly 2-oxazolines [32].

It was also observed that the drug loading is pH-dependent, and nanoparticles prepared with PBS buffer had slightly better loading (5–20%) than those prepared with DI water. The higher drug loading found in LNPs in PBS may be because, with the increase in pH, SN-38 undergoes a structural change from a closed lactone ring to an open carboxylate form, while, at pH 6.7, both forms are in equilibrium (Figure 1). The open carboxylate form is slightly more water-soluble and, hence, may incorporate into the water channels along with the bilayers, resulting in higher drug loading.

#### 3.4.3. SN-38 Release Study

**Figure 7:**
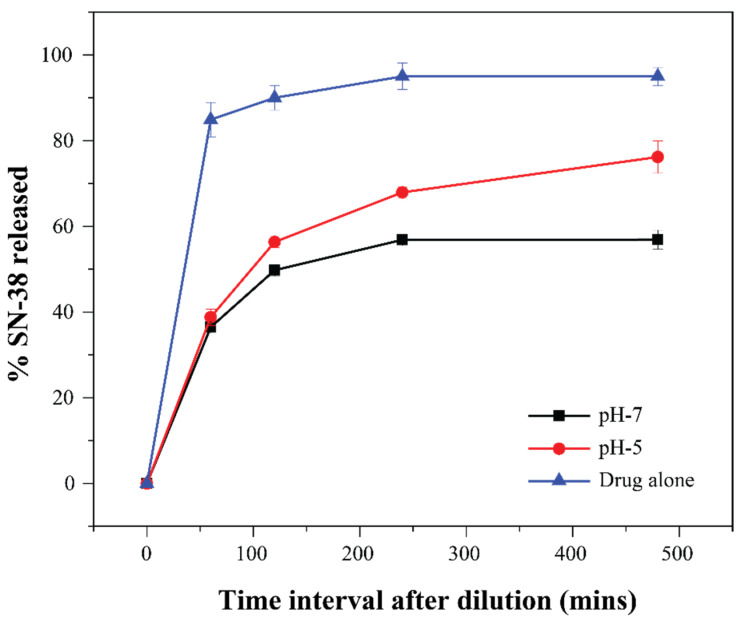
Release profile of SN-38 from the LNPs prepared using MO and OAPy-4. The LNPs were placed in a dialysis tube suspended in 1000 mL of release medium of PBS buffer at pH 7.0 and pH 5.0. SN-38-loaded nanoparticles were hexosomes at neutral pH and cubosomes at pH 5. Experiments were performed in triplicate on three separately prepared SN-38-loaded LNP formulations. All values are expressed as the mean ± SD (n = 3).

In vitro release of SN-38 from LNPs was measured using a dynamic dialysis method as described in Section 2. A control experiment with the drug alone was performed to evaluate the effect of the dialysis membrane on the release kinetics of the free drug [54]. More than 90% of free SN-38 escaped the dialysis tube within a short span of 60–90 min. The release profiles of SN-38 from the SN-38-loaded MO + OAPy-4 nanoparticles at different pH values are shown in Figure 7. The SN-38-loaded LNPs showed a burst release (~37%) for the first 60 min at both pH 7.0 and pH 5.0 and a subsequent sustained release for the next 7 h. In dialysis experiments, an ideal sink condition should be such that the solubilised drug from inside the tube should quickly cross the membrane into the sink. To achieve this, the sink volume should be 10–20 times higher than the volume required for saturated drug solution [55]. Since the solubility of SN-38 in water is 7–11 μg/mL, we used 1000 mL of sink solution for 1 mL of LNP solution. This 1000-fold dilution may be the reason for the burst release of SN-38 from the LNP, as such a dilution increases the diffusion-controlled drug release rate [56]. The burst release of the highly lipophilic drug upon dilution was similarly observed for other molecules in previous studies on hexosomes and cubosomes [57,58,59].

After the first hour of the study, compared to the samples at pH = 7.0, the drug release rate from the LNPs at pH = 5.0 was faster. This difference in sustained release behaviour is consistent with the presence of different mesophases under different pH conditions (H_2_ at pH 7.0 and Q_2_ at pH 5.0). Previous studies have demonstrated that diffusion-dependent drug release is influenced by the mesophase structure and the diameter of the water channels [60]. The faster release of SN-38 from the Q_2_ phase than the H_2_ phase can be associated with the differences in the geometry of the water channels. A cubic phase consists of two aqueous water channels, which penetrate throughout the matrix and are open to the external water phase, providing a route for the drug to escape [61]. In the case of the H_2_ phase, the aqueous compartments are closed columnar extended micellar structures, making it more difficult for drugs to diffuse to the external aqueous phase [28,62,63]. Our results agree with a previous study reported by Lee et al., who studied the in vitro release kinetics and in vivo absorption rate of three different drugs using phytantriol cubic and hexagonal bulk phases [64]. The study reported faster release kinetics in the Q_2_ phase than the H_2_ phase. Additionally, the in vivo absorption rate followed the in vitro release study trend in a rat model [64].

Previous in vitro and in vivo studies have shown that altering the release rate is one possible way to increase drug distribution to the tumour and improve the antitumour effect of the drug [36,37]. Release results for the SN-38-loaded LNPs revealed a slower drug release rate in the physiological environment (pH = 7.4) and a faster release rate in a simulated tumour microenvironment (pH = 5.0). The slower release rate at pH 7.4 is potentially beneficial because it decreases drug loss in circulation and increases the bioavailability of a drug at the tumour site. The faster release rate at pH = 5.0 is beneficial, as this leads to accumulation of the drug in pathological environments with reduced cytotoxicity for healthy cells and improved efficacy.

## 4. Conclusions

In this study, we reported the synthesis of novel pH-responsive LNPs for encapsulating and controlling the release of SN-38. We developed MO-based pH-responsive LNPs using novel synthetic ionisable aminolipids. For this purpose, four ionisable aminolipids with an amide linker were synthesised. The LNPs were formulated by doping the aminolipid into MO. These aminolipids are weak bases, which are uncharged with smaller headgroup at neutral pH; hence, incorporating them into MO at neutral pH induces the formation of hexosomes. As the pH decreases, the aminolipids become protonated with a larger effective headgroup, causing a phase transition. Among the four aminolipid-doped MO LNPs, systems containing OAPy-4 and OAPi-1 displayed a pH-dependent phase transition of hexosomes to cubosomes at pH between pH 5.0 and pH 7.0, a pathologically relevant pH range found in solid tumours.

The pH-responsive MO + OAPy-4 nanoparticles were chosen to encapsulate a very poorly soluble cancer drug, SN-38. The formulations were prepared by adding 1, 2, 5, and 10 wt.% SN-38 to the total quantity of lipid mixture in the formulation. The hydrodynamic diameter of the SN-38-loaded LNPs was in a range of 244–274 nm with a PDI of 0.19–0.26. SAXS patterns demonstrated that the addition of SN-38 did not alter the mesophase structure at neutral pH. However, the H_2_ → Q_2_ phase transition pH was shifted up by ca. 0.5 pH units in LNPs loaded with 5 and 10 wt.% SN-38 compared to the control without the drug. The drug loading experiment indicated encapsulation of 844 µg/mL of SN-38 in the prepared MO-LNPs, which was ~100 times the aqueous solubility of SN-38. Furthermore, the release study demonstrated that these highly loaded hexosomes were slow-releasing at pH 7.0 and transitioned into fast-releasing cubosomes at lower pH (pH 5).

It is worth reiterating the fact here that SN-38 is >1000 fold more potent than the commercially available prodrug, irinotecan. Currently, however, SN-38 cannot be administered directly due to extreme hydrophobicity and very poor aqueous solubility. Our study reported novel LLC lipid nanocarriers with notably high drug loading and the ability to control drug release in response to environmental pH. This study identifies significant prospects for delivering the potent SN-38 to tumour sites and suggests that the LNP delivery platform may be a viable alternative to the currently used irinotecan. The preliminary results presented here are promising; however, further evaluations are required including but not limited to long-term stability, as well as in vitro and in vivo bioactivity.

## Figures and Tables

**Table 1:** Hydrodynamic diameter of nanoformulations prepared by adding aminolipids to MO dispersed with stabiliser Pluronic F-127. The amount of aminolipid added to the formulation is represented by R_AL_, which is defined as the wt./wt. ratio of aminolipid to total lipid. Measurements were averaged from triplicates, and the results are reported as the mean ± standard deviation.

Particle Size in nm				
R_AL_	OAPy-4	OAPy-2	OAMo-1	OAPi-1
0.05	217 ± 6	284 ± 2	214 ± 4	180 ± 3
0.1	224 ± 3	255 ± 4	218 ± 3	176 ± 4
0.15	259 ± 8	300 ± 7	239 ± 3	168 ± 4
0.2	271 ± 4	267 ± 4	250 ± 2	185 ± 6
0.25	250 ± 3	313 ± 4	191 ± 8	180 ± 2
0.3	246 ± 4	290 ± 4	206 ± 11	194 ± 5
0.4	244 ± 6	272 ± 5	205 ± 6	200 ± 5
0.5	265 ± 4	300 ± 4	137 ± 6	209 ± 5

**Table 2:** Particle size distribution as expressed by polydispersity index (PDI) for nanoformulations prepared by adding aminolipids to MO dispersed with stabiliser Pluronic F-127. The amount of aminolipid added to the formulation is represented by R_AL_, which is defined as the wt./wt. ratio of aminolipid to total lipid. Data are reported as the mean ± standard deviation (n = 3).

PDI				
R_AL_	OAPy-4	OAPy-2	OAMo-1	OAPi-1
0.05	0.16 ± 0.03	0.23 ± 0.04	0.23 ± 0.03	0.25 ± 0.03
0.1	0.21 ± 0.02	0.18 ± 0.03	0.20 ± 0.05	0.20 ± 0.04
0.15	0.2 ± 0.05	0.20 ± 0.03	0.20 ± 0.05	0.23 ± 0.04
0.2	0.19 ± 0.03	0.23 ± 0.02	0.30 ± 0.03	0.22 ± 0.05
0.25	0.39 ± 0.03	0.28 ± 0.02	0.25 ± 0.03	0.19 ± 0.03
0.3	0.22 ± 0.02	0.20 ± 0.02	0.25 ± 0.05	0.23 ± 0.03
0.4	0.24 ± 0.03	0.10 ± 0.02	0.40 ± 0.04	0.15 ± 0.05
0.5	0.25 ± 0.04	0.21 ± 0.03	0.38 ± 0.03	0.18 ± 0.05

**Table 3:** EE%, drug loading (DL), and DL% for LNPs prepared by adding 1, 2, 5, and 10 wt.% SN-38 to the total quantity of lipid mixture of MO + OAPy-4, which was stabilised with Pluronic F-127 and dispersed in PBS buffer. Experiments were performed in triplicate on three independent SN-38-loaded LNP systems and all values are expressed as the mean ± SD (n = 3).

Formulation	EE%	Drug Loading (µg)	DL%
**SN-38 (1%)**	82 ± 13	172 ± 24	0.78
**SN-38 (2%)**	74 ± 11	297 ± 35	1.35
**SN-38 (5%)**	51 ± 18	516 ± 92	2.55
**SN-38 (10%)**	42 ± 14	844 ± 119	3.83

## Supplementary Materials

The following supporting information can be downloaded at https://www.mdpi.com/article/10.3390/pharmaceutics14102175/s1: Figure S1. Proton NMR spectra for synthesised ionisable amino lipids. (A) _1_H^1^ spectra for OAPy-4 (B) _1_H^1^ spectra for OAPy-2 (C) _1_H^1^ spectra for OAMo-1 and (D) _1_H^1^ spectra for OAPi-1.; Figure S2. Representative cryo-TEM images of lipid nanoparticles consisting of (A) MO-F127, (B) MO- OAPy-4 -F127 at pH 7, and (C) MO- OAPy-4 -F127 at pH 5. The ratio of OAPy-4 to MO in these formulations is 0.4.
