# Supplementary material for: Novel pH-Responsive Cubosome and Hexosome Lipid Nanocarriers of SN-38 Are Prospective for Cancer Therapy
Source: Pharmaceutics. 2022 Oct 12;14(10):2175. doi: 10.3390/pharmaceutics14102175 (PMC9609773; doi:10.3390/pharmaceutics14102175)
Supplement: Supplementary file 1 [file pharmaceutics-14-02175-s001.zip › pharmaceutics-1922076-supplementary.pdf]

Supplementary Information

Novel pH-responsive cubosome and hexosome lipid nanocarriers of SN-38 are prospective for cancer therapy

Sarigama Rajesh, Jiali Zhai, Calum J. Drummond*, Nhiem Tran*

School of Science, STEM College, RMIT University, Melbourne, VIC 3000, Australia

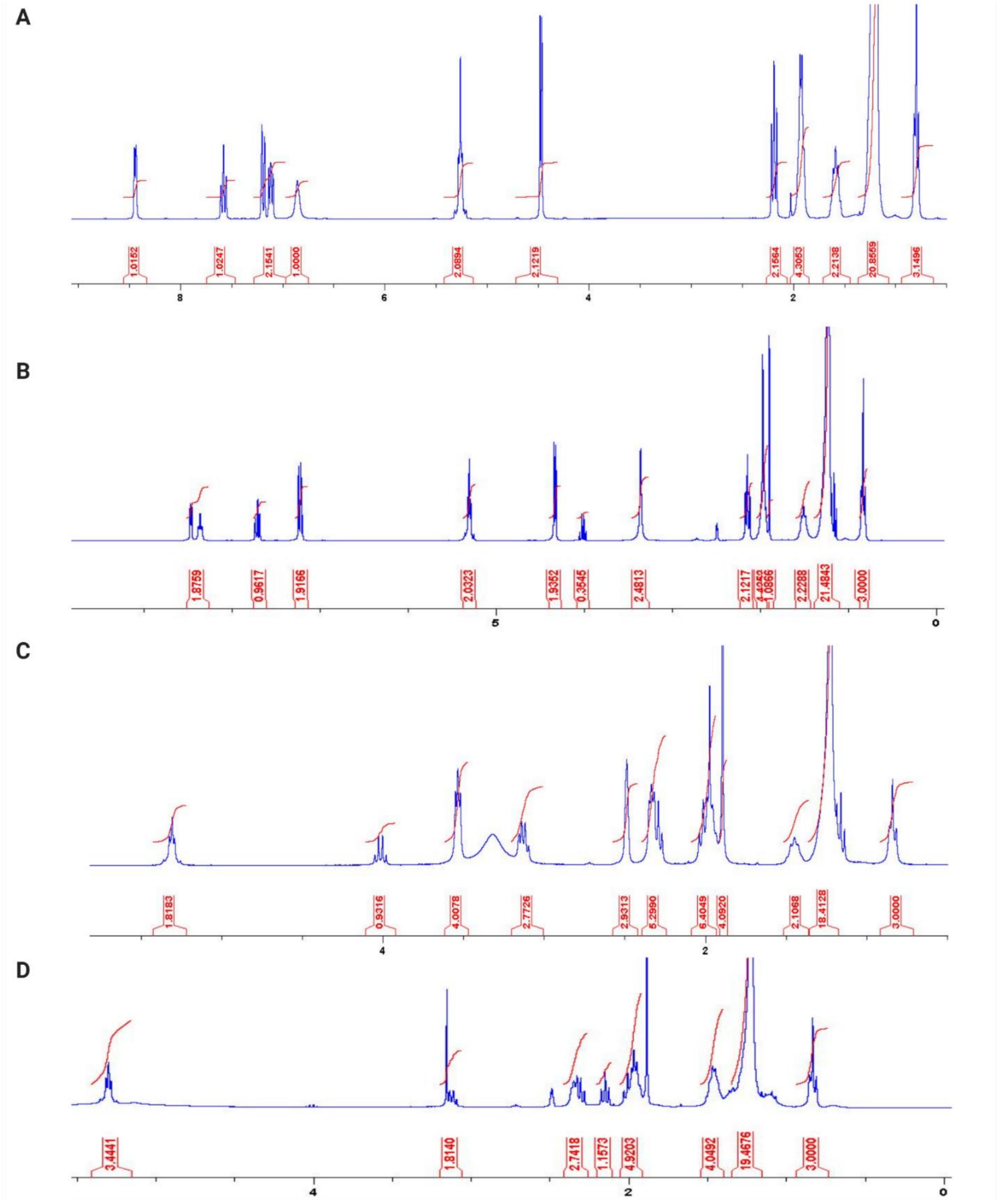

Figure S1. Proton NMR spectra for synthesised ionisable amino lipids. A) ${}_{1}H^{1}$ spectra for OAPy-4 B) ${}_{1}H^{1}$ spectra for OAPy-2 C) ${}_{1}H^{1}$ spectra for OAMo-1 and D) ${}_{1}H^{1}$ spectra for OAPi-1.

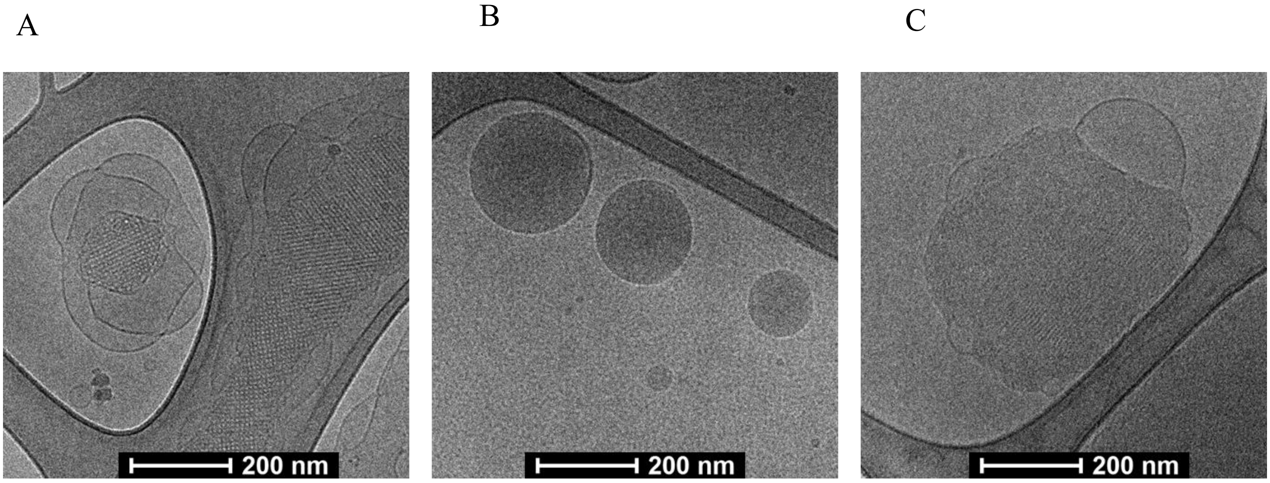

Figure S2. Representative cryo-TEM images of lipid nanoparticles consisting of (A) MO-F127, (B) MO- OAPy-4 -F127 at pH 7, and (C) MO- OAPy-4 -F127 at pH 5. The ratio of OAPy-4 to MO in these formulations is 0.4.
